# Anticancer Properties and Mechanisms of Fucoidan on Mouse Breast Cancer In Vitro and In Vivo

**DOI:** 10.1371/journal.pone.0043483

**Published:** 2012-08-20

**Authors:** Meilan Xue, Yinlin Ge, Jinyu Zhang, Qing Wang, Lin Hou, Yongchao Liu, Lingling Sun, Quan Li

**Affiliations:** 1 Department of Biochemistry and Molecular Biology, Medical College, Qingdao University, Qingdao, China; 2 Department of Ophthalmology, The Affiliated Hospital of Qingdao University, Qingdao, China; 3 Department of Pathology, The Affiliated Hospital of Qingdao University, Qingdao, China; University of South Alabama, United States of America

## Abstract

**Background:**

Fucoidan is a sulfated polysaccharide derived from brown algae that has been reported to perform multiple biological activities, including antitumor activity. In this study, we examined the influence of crude fucoidan on mouse breast cancer in vitro and in vivo.

**Materials and Methods:**

In vitro, fluorescent staining, flow cytometry and Western blot were performed to analyze apoptosis and vascular endothelial growth factor (VEGF) expression of mouse breast cancer 4T1 cells. In vivo, therapy experiments were conducted on Babl/c mice bearing breast cancer. The tumor volume and weight were measured. The number of apoptotic cells and microvascular density (MVD) in tumor tissues were assessed by TUNEL and CD34 immunostaining. Immunohistochemical assays and ELISA assay were used to detect the expression of VEGF in tissues.

**Results:**

In vitro studies showed that crude fucoidan significantly decreased the viable number of 4T1 cells, induced apoptosis and down-regulated the expression of VEGF. The expression of Bcl-2 was decreased, and the ratio of Bcl-2 to Bax was significantly decreased. The expression of Survivin and phosphorylated extracellular signal regulated protein kinases (ERKs) was decreased. Cytochrome C was released from mitochondria into cytosol, and the cleaved Caspase-3 protein rose after fucoidan treatment. Intraperitoneal injection of fucoidan in breast cancer models reduced the tumor volume and weight. The enhanced antitumor efficacy was associated with decreased angiogenesis and increased induction of apoptosis.

**Conclusion:**

These findings indicated that crude fucoidan inhibited mouse breast cancer growth in vitro and in vivo. These data suggest that fucoidan may serve as a potential therapeutic agent for breast cancer.

## Introduction

Fucoidan is a complex sulfated polysaccharide that is found in brown algae. The structures and compositions of fucoidan vary among different brown seaweed species, but generally the compound primarily consists of L-fucose and sulfate, along with small quantities of D-galactose, D-mannose, D-xylose, and uronic acid [1]–[3]. Recently, the diverse biological activities of fucoidan have been studied intensively; the putative bioactivities of fucoidan include antioxidant [4] and immunomodulatory, antivirus, antithrombotic and anticoagulant effects [5]–[8]. In particular, the antitumor activity has recently attracted considerable attention. There have also been a variety of studies addressing the anticarcinogenic effects of fucoidan. Fucoidan has been reported to enhance the activity of NK (natural killer) cells which is an important factor in anti-cancer activity [6]. In previous in vivo studies conducted using xenograft models, fucoidan has been reported to suppress the growth of Ehrlich ascites carcinoma [9], [10] and Lewis lung adenocarcinoma, and has also been shown to inhibit the metastasis of Lewis lung adenocarcinoma [5] and 13762 MAT rat mammary adenocarcinoma [11]. The findings of previous in vitro studies have demonstrated that fucoidan inhibits the growth of non-small-cell bronchopulmonary carcinoma NSCLCN6 cells [12] and human lymphoma HS-Sultan cells [13], and also inhibits the invasion of HT1080 human fibrosarcoma cells and the angiogenic activity of HeLa human uterine carcinoma cells [14]. Recently, fucoidan has been reported to induce apoptosis in several cancer cell lines [5], [15], Fucoidan has also been shown to induce a substantial reduction in viable cell numbers and apoptosis of human lung carcinoma A549 cells as well as colon cancer HT-29 and HCT116 cells in a dose-dependent manner [16], [17]. But the mechanism is controversial because it is uncertain which cascade plays a pivotal role in the induction of apoptosis by fucoidan.

However, to the best of our knowledge, the effects of crude fucoidan on the growth of breast cancer bearing mice and its underlying mechanisms have not yet to be determined in detail. Therefore we examined the effects of crude fucoidan extracted from F. vesicu-losus on the growth of breast cancer in vitro and in vivo, and to determine the mechanisms relevant to this effect.

## Results

### Effect of fucoidan on the growth of normal mouse fibroblasts

**Figure 1:**
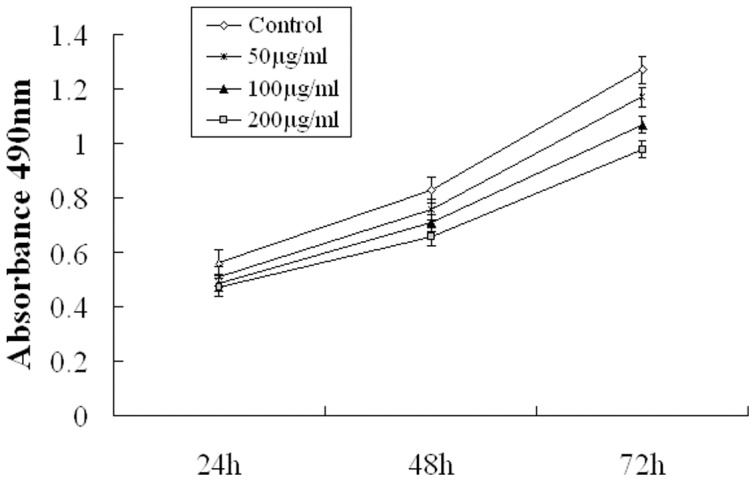
Effect of fucoidan on the growth of normal mouse fibroblasts. The effect of fucoidan on the proliferation of normal mouse fibroblasts (L929 cells) was measured by MTT assay. The cells viability of each fucoidan treatment group(50,100 and 200µg/ml) was not statistically significant, compared with that of untreated controls (*P*>0.05). Data are shown as mean ± S.D. Fucoidan did not affect the viability of normal mouse fibroblasts.

The effect of fucoidan on the proliferation of normal mouse fibroblasts (L929 cells) was measured by MTT assay. As shown in Fig. 1, the cells viability of each fucoidan treatment group(50,100 and 200 µg/ml) was not statistically significant, compared with that of untreated controls (*P*>0.05). This result showed that fucoidan did not affect the viability of normal mouse fibroblasts.

### Fucoidan inhibited the growth of 4T1 cells

**Figure 2:**
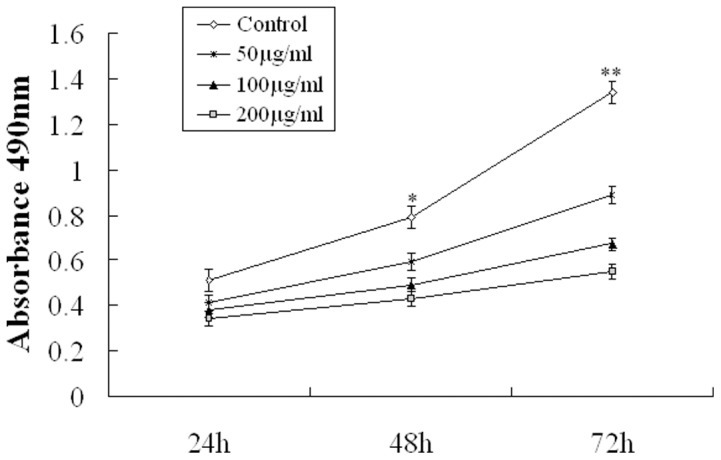
Inhibitory effect of fucoidan on the growth of 4T1 Cells. The 4T1 cells were treated with 50, 100, and 200 µg/ml fucoidan separately for 1d, 2d and 3 d and measured for viability by MTT assay. Data are shown as mean ± S.D. n = 5. *P<0.05, vs. Control, ** *P*<0.01. vs. Control.

In vitro assessment of the effects of different concentrations (50,100 and 200 µg/ml) of crude fucoidan on the growth of 4T1 cells was performed via measurement of cell viability using an MTT assay. The effect of various doses of fucoidan on 4T1 cells viability was shown in Fig. 2. 4T1 cells grown in control media displayed a continuous increase in viable cell number over the 3-day culture period. These results indicated that treatment with 50, 100 and 200 µg/ml fucoidan decreased the 4T1 cells viability in a time-dependent manner (*P*<0.05). After 3 days of culturing, 200 µg/ml fucoidan decreased viable 4T1 cell number by 85.3% as compared with those of untreated controls.

### Fucoidan induced apoptosis of 4T1 cells

**Figure 3:**
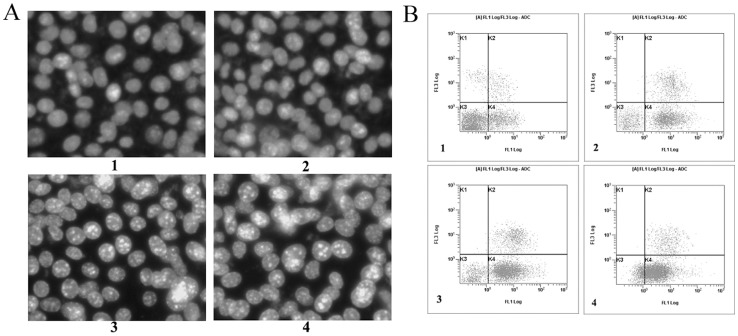
Apoptosis induction after 48 h treatment with fucoidan in 4T1 cells. A) Morphology of 4T1 cells observed under fluorescence microscope (Hoechst33258×400). DNA fragment and chromatin condensation were observed in fucoidan-treated cells, but few in untreated cells. The 50,100,200 µg/ml fucoidan treated 4T1 cells that underwent apoptosis were calculated at 18.9±1.17%, 42.3±4.38% and 67±7.52% respectively, while there were7.4±0.29% of the apoptotic cells in the control cells (*P*<0.01). B) The apoptosis cells determined by FCM. 4T1 cells were stained with annexin V-FITC and propidium iodide (PI). The lower right quadrant (annexin V+/PI−) represents early apoptosis, and the upper right quadrant (annexin V+/PI+) represents late apoptosis and necrosis. The early apoptotic rates of 4T1 cells treated with fucoidan were 24±1.34%, 41±3.75% and 59±5.27% respectively and significantly higher than that in control cells(11±0.89%) (*P*<0.05). Data are a representative of three independent experiments showing similar results. 1: control; 2: fucoidan 50 µg/ml; 3: fucoidan 100 µg/ml; 4: fucoidan 200 µg/ml.

An apoptotic cell has special morphologic features such as cell shrinkage, chromatin condensation and margination as well as forming apoptotic bodies. In order to determine whether the fucoidan-inducing reduction in cell viability was attributable to the induction of apoptosis, we stained the 4T1 cells with Hoechst 33258 dye. The treatment of 4T1 cells with fucoidan resulted in the induction of chromatin condensation and fragmentation, which could be visualized as intense pycnotic bluish-white fluorescence within the cell nuclei. The 50,100,200 µg/ml fucoidan treated 4T1 cells that underwent apoptosis were calculated at 18.9±1.17%, 42.3±4.38% and 67±7.52% respectively, while there were 7.4±0.29% of the apoptotic cells in the control cells (*P*<0.01) (Fig. 3A). We subsequently estimated the number of apoptotic cells by flow cytometry. The proportionof apoptotic increased in a concentration-dependent manner in cells that had been treated with fucoidan (Fig. 3B). When treated with fucoidan (50,100,200 µg/ml) for 48 h, the early apoptotic rates of 4T1 cells were 24±1.34%, 41±3.75% and 59±5.27% respectively and significantly higher than that in control cells(11%±0.89%) (*P*<0.05).

### Fucoidan altered the levels of the Bcl-2, Survivin, and phosphorylated ERKs

**Figure 4:**
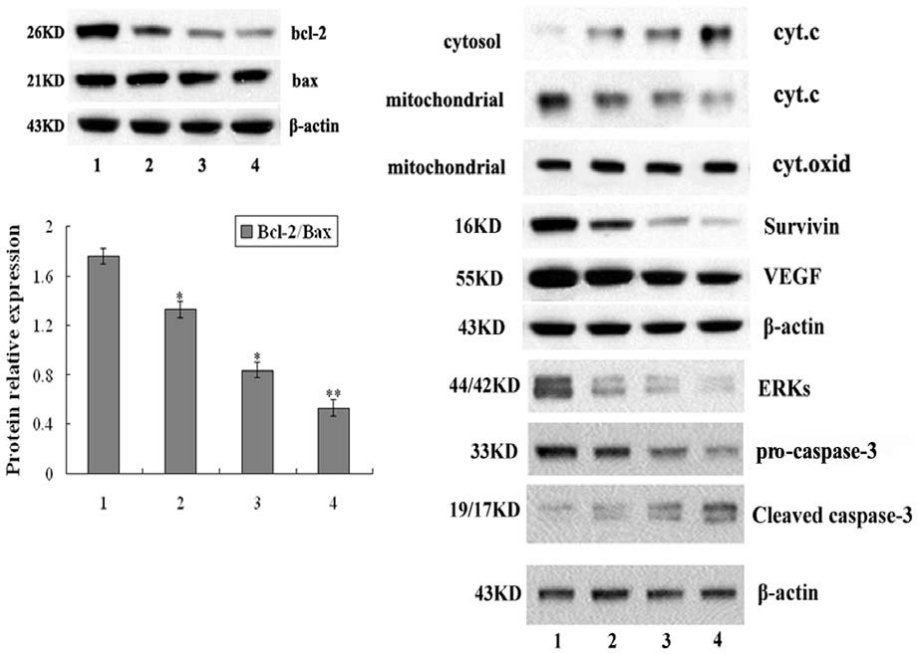
Expression of Bcl-2, Bax, Survivin, ERK, cytochrome C, Caspase-3 and VEGF in 4T1 cells after 48 h treatment of fucoidan detected by Western blot. Expression of the β-actin gene was used as the internal control. Data are mean±SD of one representative experiment performed in triplicate. Fucoidan significantly decreased the expression of Bcl-2, did not modulate the expression of Bax, but the ratio Bcl-2/Bax was decreased. Fucoidan also decreased the protein expression of Survivin and phosphorylated ERKs in 4T1 cells. Fucoidan treatment significantly increased levels of cytochrome C in the cytoplasm, whereas there was a significant corresponding decline of mitochondrial cytochrome C. Cytochrome oxidase (subunit II) was as sample loading control (cyt. oxid). The level of pro-caspase-3 decreased after fucoidan treatment, while the level of caspase-3 cleavage protein increased after fucoidan treatment. Fucoidan decreased VEGF expression in 4T1 cells. Lane 1: control cells; lanes 2–4: 4T1 cells treated with 50, 100 and 200 μg/ml fucoidan respectively. *P<0.05, vs. Control; **P<0.01, vs. Control.

To investigate the possible mechanism underlying the induction of apoptosis by fucoidan, we examined the expression of Bcl-2 and Bax in 4T1cells after treatment with fucoidan. Bcl-2 family proteins play critical roles in the regulation of apoptosis by either pro-apoptotic such as Bax, or anti-apoptotic such as Bcl-2. The ratio of the pro- and anti-apoptotic molecules was essential to apoptosis. The result of Western-blotting showed that fucoidan significantly decreased the expression of Bcl-2, did not modulate the expression of Bax (Fig. 4), but the ratio Bcl-2/Bax was decreased. This result demonstrated fucoidan has proapoptotic effects on 4T1 cells.

Survivin is a unique member of the IAP (inhibitors of apoptosis) family. To examine whether fucoidan can affect Survivin expression, we analyzed the levels of survivin protein using Western blot. As shown in Fig. 4, fucoidan decreased the protein expression of survivin in 4T1 cells. Western blot analysis indicated phosphorylated ERKs expression was down-regulated when 4T1 cells were treated with fucoidan (50, 100, 200 μg/ml) for 48 h.

### Fucoidan induced cytochrome C release and up-regulated Caspase-3 protein expression

In biological system, apoptosis may involve disruption of mitochondrial function through the abnormal expression of Bcl-2 and/or Bax which induces the release of cytochrome C from mitochondria into the cytosol. Cytosolic cytochrome C can lead to the activation of caspases in the apoptosome and finally lead to the activation of caspase-3. Activation of caspase-3 subsequently leads to apoptosis. Therefore, we determined whether induction of apoptosis in 4T1 cells by fucoidan is associated with disruption of mitochondrial function and activation of caspases. Fucoidan treatment significantly increased levels of cytochrome C in the cytoplasm (Fig. 4).

Activated or cleaved caspase-3 (17 kDa and 19 kDa) is crucial for the induction of apoptosis. The level of pre-caspase-3 decreased significantly after fucoidan treatment, while the level of caspase-3 cleavage protein increased after fucoidan treatment. The result was shown in Fig. 4. Increase in the Caspase-3 activity that arises after the cleavage of pro-caspase-3 is a hallmark of induced apoptosis and is considered to be a point of no return. These data demonstrated that fucoidan induced caspase-3-dependent apoptotic pathway in 4T1 cells.

### Fucoidan down-regulated VEGF expression

In order to investigate whether fucoidan has potent anti-angiogenic activity, we examined the VEGF expression in 4T1 cells after treatment with fucoidan by Western-blotting. The expression of VEGF protein was significantly suppressed by fucoidan (*P*<0.05) (Fig. 4).

### Fucoidan inhibits 4T1 tumor growth in vivo

**Figure 5:**
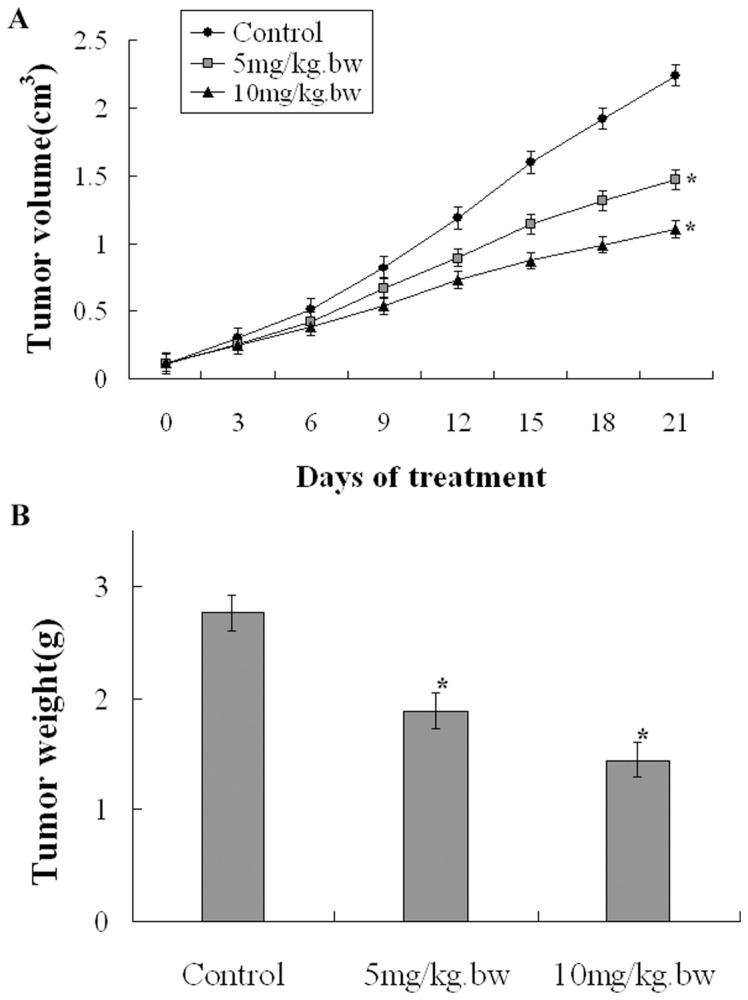
Antitumor effects of fucoidan on breast cancer in vivo. Fucoidan reduced the tumor volume and the tumor weight in Balb/c mice. A) Tumor growth curves. Each point in the curves represents the mean ± SD (n = 7 tumors). The therapy started when the tumors reached a volume of 0.1cm^3^. The fucoidan inhibited tumor growth, *P<0.05 vs Control. B) Weight of the tumors. Each bar represents the mean ± SD (n = 7 tumors). Mean weights of the tumors are 2.77g, 2.15g, and 1.19g, for the control group, 5 mg/kg.bw and 10mg/kg.bw fucoidan group respectively. *P<0.05 vs Control.

The prominent inhibitory effect of fucoidan on 4T1 cell proliferation in vitro suggested that it might suppress tumor growth in vivo. To verify this hypothesis, we s.c. inoculated female Balb/c mice with 4T1 cells. After 10 days, visible tumors had developed at the injection sites (8∼10 mm^2^ in size). Then 4T1 tumor bearing Balb/c mice were treated by intraperitoneal injection of 5 and 10 mg/kg.bw fucoidan and 0.1 ml NS alone (as blank control), and in each treatment seven animals were used. The whole process were repeated every 2 days and were conducted for totally ten times. As shown in Fig. 5, fucoidan reduced the tumor volume and the tumor weight in Balb/c mice.

### Fucoidan induced apoptosis in tumor tissue

**Figure 6:**
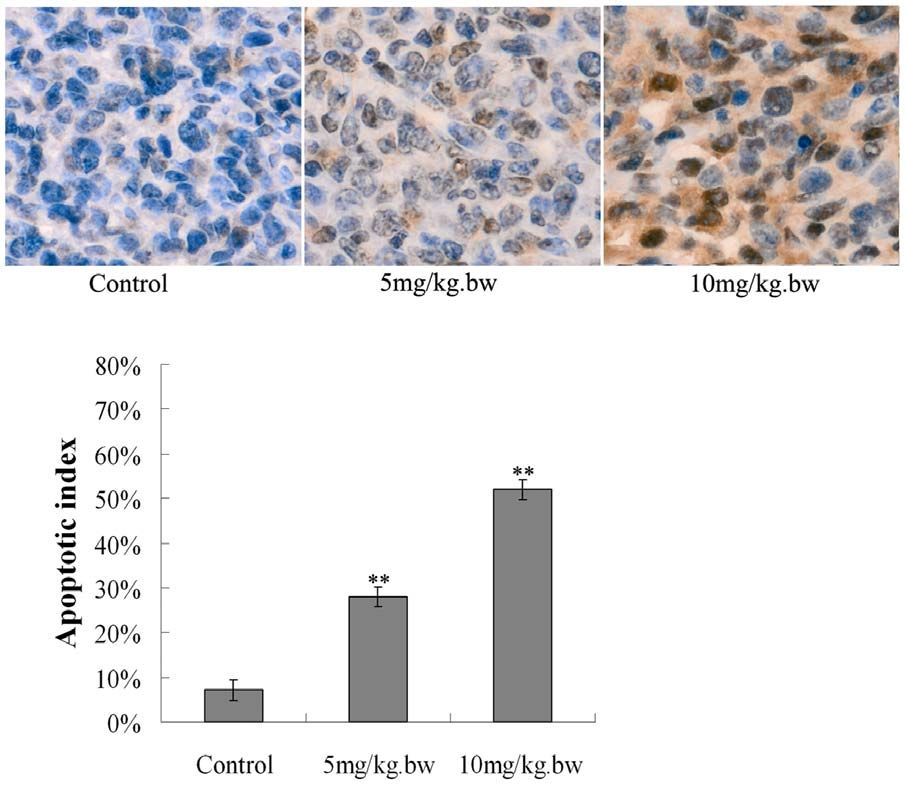
Fucoidan induced apoptosis in vivo. Representative photographs of the tumor sections examined by TUNEL assay. TUNEL-positive cell nuclei (dark brown) were observed under a fluorescence microscope (×400). The number of apoptotic cells was counted 5 random fields (×400) in a blinded manner. Each bar represents the “apoptosis index”, expressed as mean ± SD. Fucoidan treatment caused a significantly higher percentage of TUNEL-positive apoptotic cells. **P<0.01 vs Control.

To further confirm the ability of fucoidan to elicit apoptosis in vivo, in situ TUNEL staining was carried out on tissue sections of tumors excised on the 20^th^ day since 4T1 cell-implanted mice were treated with various doses of fucoidan. As illustrated in Fig. 6, fucoidan treatment caused a significantly higher percentage of TUNEL-positive apoptotic cells(*P*<0.01). These results indicated that fucoidan could exert a strong antitumor effect on breast cancer model and is a potent apoptosis-inducing agent in vivo.

### Fucoidan inhibited angiogenesis in tumor tissue

**Figure 7:**
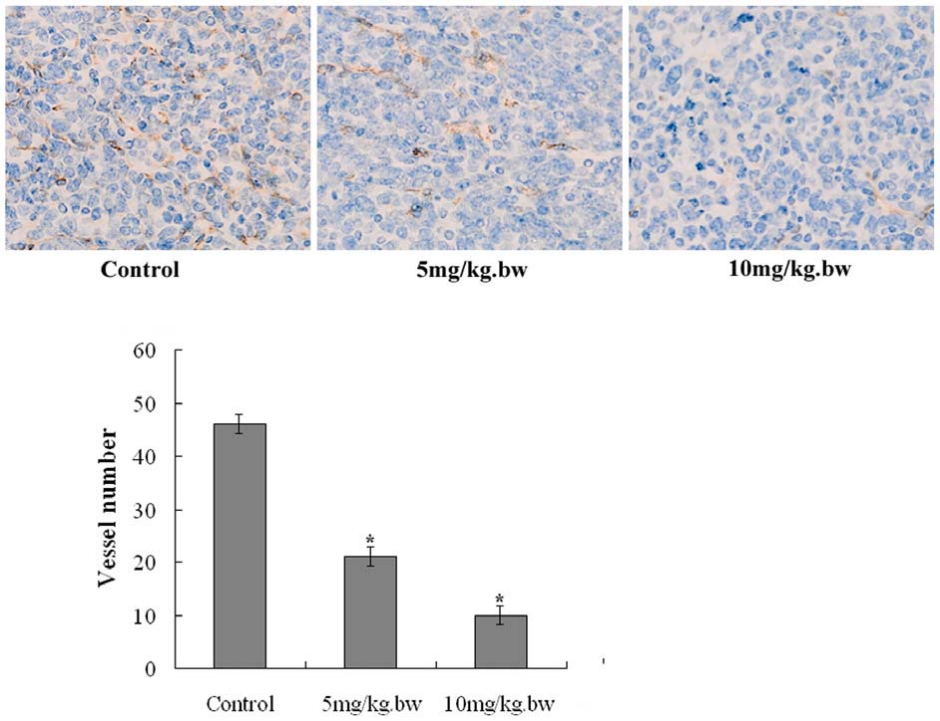
Fcoidan inhibited the tumor angiogenesis in vivo. Representative photographs of the tumor sections examined by immunohistochemical staining for CD34 showing tumor vasculature (×400). The microvessel number was reduced apparently in the tumors belonging to the mice treated with fucoidan. Each bar represents the average vessel number for each group, expressed as mean ± SD. *P<0.05 vs Control.

**Figure 8:**
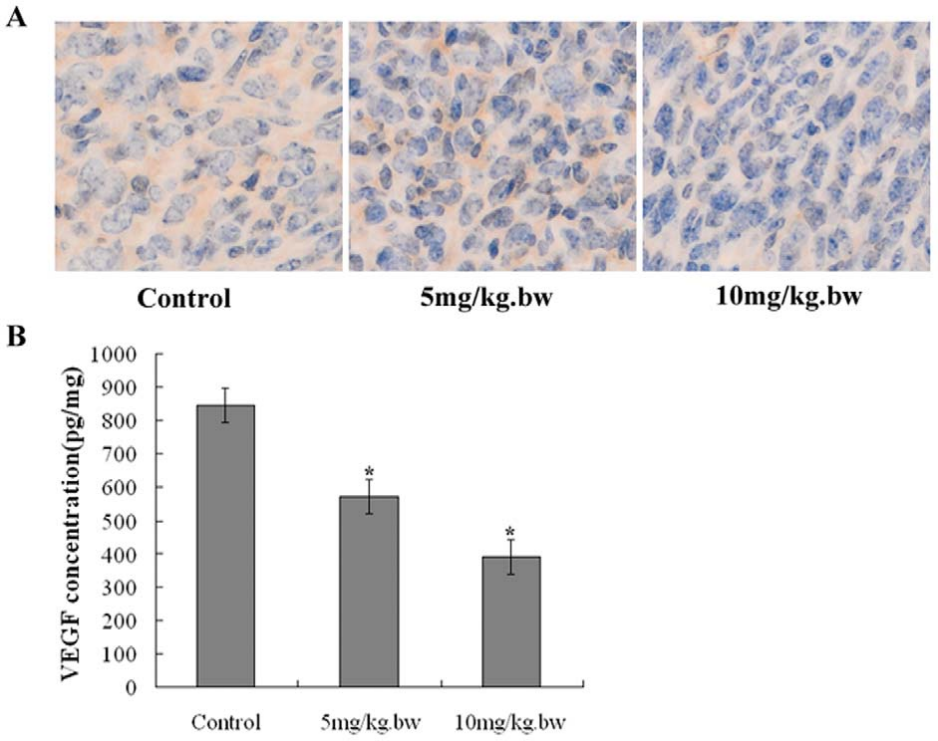
Fucoidan down-regulated VEGF expression in vivo. A) Representative photographs of the tumor sections examined by immunohistochemical staining for VEGF (×400 magnification). The assessment of VEGF was based on a cytoplasmic staining pattern. B) The tumors were harvested and assayed for VEGF protein by ELISA. The results are presented as mean ± SD (n = 7 tumors). Fucoidan caused significant reduction in intratumoral VEGF expression compared with control group. *P<0.05 vs Control.

To investigate the effect of fucoidan on angiogenesis in vivo, we evaluated microvessel density in the harvested tumors. As shown in Fig. 7, the microvessel number was apparently reduced in the tumors belonging to the mice treated with fucoidan(*P*<0.05). Moreover, we harness the tumors for immunohistochemistry and ELISA assay to measure the expression of VEGF. As shown in Fig. 8A, we analyzed the gross distribution of immunoreactive VEGF in the tumors and observed a general decrease of VEGF staining in the tumors belonging to the mice treated with fucoidan, whereas the tumors of control group exhibited significantly more VEGF staining(*P*<0.05). Consistently, the ELISA assay showed that fucoidan caused a significant reduction in intratumoral VEGF expression compared with control group (*P*<0.05), as shown in Fig. 8B.

### Fucoidan suppressed lung metastasis of breast cancer

**Figure 9:**
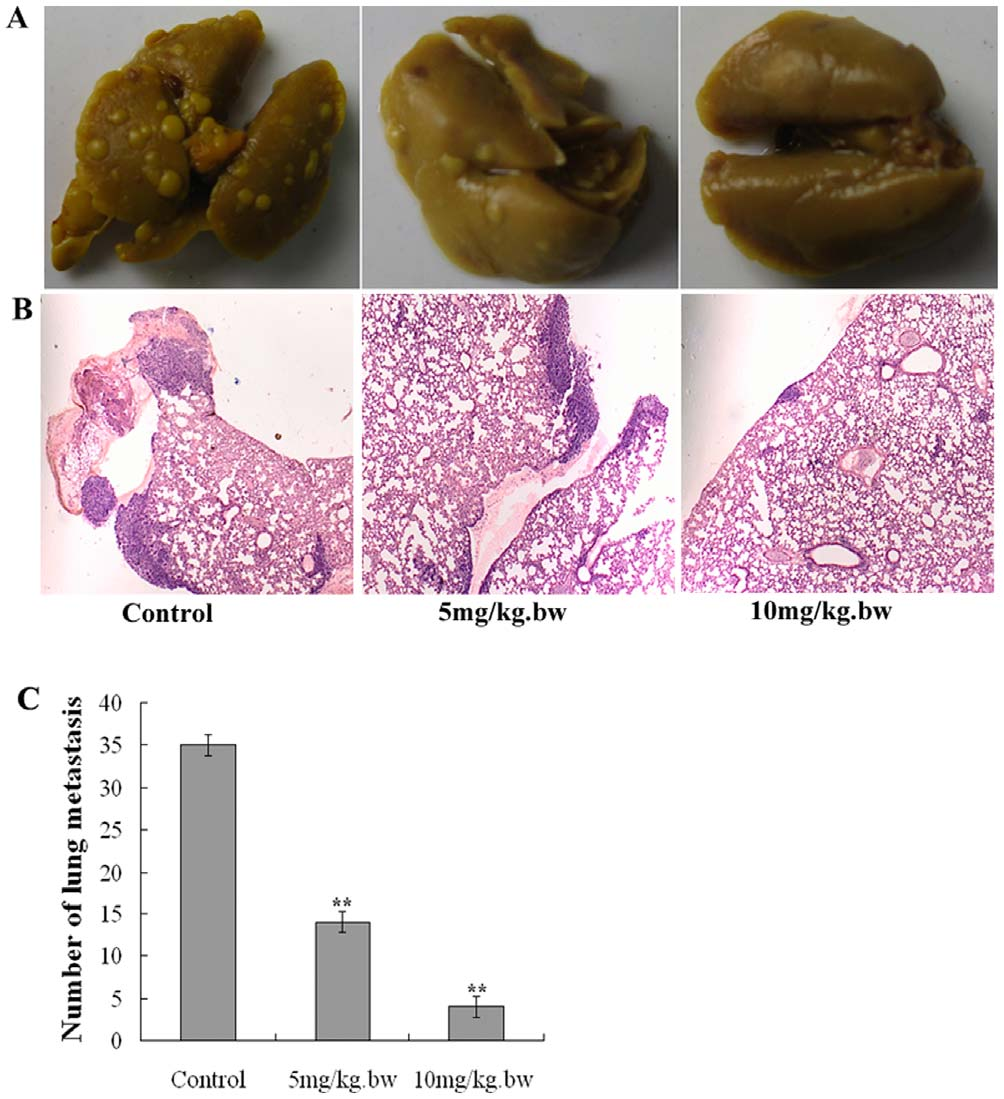
Fucoidan decreased lung metastases of breast cancer. A) Representative photographs of lung metastases after treatment of fucoidan. Mice were euthanized 20 days after treatment. Lungs were harvested, put in a fixative (saturated picric acid: formaldehyde: acetic acid  = 75:25:5) for 24 h, and then restore the color to the lung tissue by soaked in absolute alcohol. B) Photograph of Hematoxylin and eosin-stained sections in lung metastatic nodule (×20 magnification). The deep blue dye cell mass was breast cancer metastases. C)The number of lung metastatic nodules (white) were counted. The results are presented as mean ± SD (n = 7 tumors). The results are presented as mean ± SD (n = 7 tumors). Compared with control mice, fucoidan resulted in significantly fewer lung metastases. **P<0.01 vs Control.

4T1 cell is a highly invasive breast cancer cell lines. To investigate the effect of fucoidan on invasiveness of 4T1 tumor, mice were euthanized, and the lungs harvested for enumeration of lung metastatic nodules metastases after fucoidan treatment. The results are shown in Fig. 9. Compared with control mice, and who had an average of 34 nodules per mouse, fucoidan resulted in significantly fewer lung metastases (*P*<0.01).

## Discussion

Fucoidan is a complex sulfated polysaccharide obtained from naturally occurring edible brown seaweeds, which has been consumed as a dietary fiber in many Asian countries for centuries. Fucoidan has been shown to inhibit the growth of a wide variety of tumor cells [5], [18]. Therefore, fucoidan has become a focus of great interest because of its anticancerous activity and is expected to be a new candidate for low toxic cancer therapy. In this study, we used a commercial available fucoidan purified from *F. vesicu-losus*. We demonstrated that fucoidan strongly presented apoptosis induction and angiogenesis inhibition on mouse breast cancer in vitro and in vivo. However, fucoidan did not affect proliferation of normal cells. It has also been reported that fucoidan inhibited the growth of peripheral blood mononuclear cells (PBMCs) from adult T-cell leukemia patients but not that of normal PBMC [19]. Therefore, fucoidan may be a good chemopreventive and antitumor candidate without toxic effects on normal cells.

In this study we investigated in detail the molecular pathways induced by fucoidan on breast cancer. Apoptosis is a biological process requiring activation of several signaling cascades. It is widely accepted that mitochondrial pathway is one of apoptosis pathways. Pro-survival Bcl-2 and its helpers compete with Bax and other pro-apoptosis proteins to regulate the release of proteins, such as c-Jun amino-terminal kinase(JNK), and mitogen-activated protein kinase (MAPK)/extracellular signal regulated kinase(ERK), and cytochrome *C* from mitochondria, which in turn activate caspases [20]. The Bcl-2 family consists of both antiapoptotic and proapoptotic members that are important members involved in apoptosis. Bax can promote the release of cytochrome C into the cytosol from mitochondria. The antiapoptotic proteins such as Bcl-2 preserve the integrity of the mitochondria. This blocks the release of cytochrome C that activates the effectors of apoptosis [21]. Since Bcl-2 functions by forming a heterodimer with its pro-apoptotic partner Bax, the Bcl-2/Bax ratio is proportional to the relative sensitivity or resistance of the cells to various apoptotic stimuli [22]. Survivin is a unique member of the inhibitor of apoptosis (IAP) protein family and has been shown to be involved in both control of cell division and inhibition of apoptosis. Accumulating data indicate that altered expression and/or function of survivin in cancer cells are associated with cancer progression, drug/radiation resistance, poor prognosis, and short patient survival [23], [24]. In the present study, the apoptotic induction of fucoidan was also accompanied by inhibition of extracellular signal-regulated kinases (ERKs) [25]. The ERKs pathway thus may play a major regulatory role for apoptosis. Zhang Z, et al [26] found that fucoidan extract could induce phosphorylation of ERK1/2 in MCF-7 cells.

Our results indicated fucoidan reduced the Bcl-2 level, resulting in a decrease in Bcl-2/Bax ratio, down-regulation of survivin and the decrease of phosphorylated ERKs activity in 4T1 cells. These results revealed that the molecular events occurring during fucoidan-induced mitochondria-mediated apoptosis. Inhibition of the expression of anti-apoptotic factors will result in the release of cytochrome C from mitochondria and the activation of caspase-3, one of the key executioners of apoptosis. In this study, we found that treatment of fucoidan resulted in the release of cytochrome C, and finally increased expression of cleaved caspase-3. Cleaved caspase-3 results in increased apoptosis in 4T1 cells. This result was consistent with effect of fucoidan on human colon cancer cells [17]. Based on the results, we concluded that several critical links such as Bcl-2, Survivin, ERKs, cytochrome C and caspase-3 play very important roles in apoptosis of 4T1 cells induced by fucoidan. In addition, the results strongly supported the hypothesis of mitochondrial apoptotic pathway involvement.

Furthermore, fucoidan exhibited potent anti-angiogenic activity and suppressed lung metastasis of breast cancer in vivo. We observed down-regulation of VEGF proteins both in vitro and in vivo. Suppression of VEGF proteins induced by fucoidan is likely caused by interference of ERKs signaling. It has previously been demonstrated that activation of the p38 and p42/44 MAPK up-regulates VEGF and other proinflammatory cytokines [27]. Metastasis is a rather complex process that occurs through a series of steps that include invasion, intravasation, transport through the circulatory system, arrest at a secondary site, and the extravasation and growth in a secondary organ [28]. Koyanagi S, at al [29] found that over-sulfated fucoidans clearly suppressed the neovascularization induced by Sarcoma 180 cells that had been implanted in mice. VEGF can promote tumor cell metastasis by triggering cell migration and invasion in an autocrine fashion. Focoidan suppressed the mitogenic and chemotactic actions of vascular VEGF_165_ by preventing VEGF_165_ from binding with its cell surface receptor. Fucoidan resulted in fewer lung metastases, which is at least partially relevant to the down-regulation of VEGF expression. Further study is required to provide more proof.

In conclusion, fucoidan induced apoptosis, inhibited angiogenesis and suppressed lung metastasis of breast cancer in vitro and in vivo. The possible mechanism might be that fucoidan inhibits expression of Bcl-2, Survivin, ERKs and VEGF and increases activation of Caspase-3. Fucoidan might be able to improve the survival of breast cancer patients by promoting apoptosis, interfering with angiogenesis and inhibiting lung metastasis. So we strongly suggest that fucoidan be a potential therapeutic agent for the prevention and treatment of breast cancer.

## Materials and Methods

### Ethics Statement

All experimental procedures were conducted in conformity with the National Institutes of Health Guide for Care and Use of Laboratory Animals (Publication No.85–23, revised 1985). The study protocol was approved by the Review Committee for the Use of Human or Animal Subjects of Medical College of Qingdao University.

### Fucoidan

Crud fucoidan was purchased from Sigma (St. Louis, MO, U.S.A.). The fucoidan was dissolved in normal saline to 20 mg/ml and stored at −20°C until further use.

### Cell Culture

The 4T1 mouse breast cancer cells were purchased from Shanghai Life Science of Chinese Academy of Sciences. 4T1 cells were routinely maintained in 1∶1 (v/v) mixture of DMEM high glucose (Hyclone, Beijing, China)and 10% (vol/vol) fetal bovine serum (FBS) (Gibco BRL, Grand Island, NY, U.S.A.), 37°C in a tissue culture incubator with 5% CO_2_ and 98% relative humidity. The 4T1 cells were placed in 6-well plates and cultured as normal. The exponentially growing cells were used throughout the experiments.

### MTT Assay

The 3-[4,5-dimethylthiazol-2-yl]-2,5-diphenyltetrazolium bromide (MTT, Roche Diagnostics Corporation) assay was performed as previously described [30]. In brief, 4T1 cells were cultured in a 96-well plate at a density of 1×10^5^ cells per ml. The cells were then treated with 50, 100, and 200 µg/ml of fucoidan. After 1d, 2d and 3 d, the cells were treated with 20 µl MTT (5 mg/ml). The cultures were then re-incubated for an additional 4 h. After removal of the supernatant, 150 µl DMSO was added to each well to dissolve the crystals completely and then the absorbance was measured at 490 nm using an ELISA Reader (Bio-Rad, USA).

### Apoptotic cell morphology observation

Cells were seeded in 24-well plates with glass slides on the bottom of wells, and treated with 50, 100, and 200 µg/ml of fucoidan for 48 h. After the slides were washed with cold PBS gently, cells were fixed by 4% polyformaldehyde for 1 h and then washed 3 times with PBS. The resulting cells were stained with 0.5 ml Hoechst 33258 (10 µg/ml) at 37°C for 10 min in the dark. The apoptotic features of cell death were established by staining cell nuclei with the DNA-binding fluorochrome Hoechst 33258 and assessing chromatin condensation by fluorescence microscopy (BX-50, Olympus, Japan) [31]. In each group, 5 microscopic fields were randomly selected and five hundreds cells were counted. Apoptotic cell was then calculated as the percentage of apoptotic cells over the total number of blue fluorescent protein-positive cells.

### Flow Cytometric Analysis

The annexin V-FITC apoptosis detection kit was used for the apoptosis assay (Invitrogen). 4T1 cells (1×10^6^ cells/ml) were treated with 50, 100, and 200 µg/ml of fucoidan for 48 h. Cells were harvested by trypsinization, washed twice with PBS, and resuspended in 500 μL of binding buffer. Cell suspensions were then incubated with 5 μL of annexin V-FITC and 5 μL of propidium iodide (PI) for 10 minutes at room temperature in the dark. The cells were evaluated immediately by ﬂow cytometry (Coulter, Becton Dickinson, USA).

### Western blot analysis

4T1 cells were seeded in 6-well plates and treated with 50, 100, and 200 µg/ml fucoidan. After 48 h, with 2% SDS (10mM EDTA, 50 mM Tris base, 10% SDS, pH 8.0) and boiled at 100°C for 10 minutes. Protein concentrations were measured using the BCA protein assay. Briefly, 50 μg samples of protein were loaded on 12% SDS-PAGE gels, transferred to PVDF membranes and blocked with 5% nonfat milk in TBST buffer (20 mM Tris-HCl, 120 mMNaCl, 0.1% Tween for 1 hour [32]. Membranes were incubated with various primary antibodies against Bcl-2, Bax, Caspase-3, Survivin, VEGF, and phospho-p42/p44 extracellular signal regulated protein kinases (ERKs) at 4°C overnight. The rabbit polyclonal antibody for β-catenin, VEGF, and bcl-2, mouse monoclonal antibody for bax and ERKs, and rabbit monoclonal antibody for survivin were purchased from Santa Cruz Biotechnology (Santa Cruz, CA, USA). After washing, the blots were incubated with peroxidase-conjugated donkey anti-rabbit or anti-mouse secondary antibody for 2 hours. Protein expression was quantified with a Gel EDAS analysis system (Cold Spring USA Corporation) and Gel-Pro Analyzer 3.1 software (Media Cybernetics).

### Assay for mitochondrial cytochrome C release

This assay was performed according to cytochrome C releasing apoptosis assay kit's (Biovision, USA) instructions. In brief, after treatment, 1×10^6^ cells were pelleted by centrifugation and washed twice with ice-cold PBS. Cell pellets were resuspended with 1 ml cytosol extraction buffer mix containing DTT and protease inhibitor, and incubated for 10 min on ice. After homogenization, unbroken cells and large debris were removed by centrifugation. The resulting supernatants were saved as cytosolic extracts at −70°C. The pellets were resuspended with 100 μlextraction buffer mix containing DTT and protease inhibitor, and saved as mitochondrial fractions. Load 30 μg cytosolic and mitochondrial fractions isolated from 4T1 cells on 12% SDS-PAGE. Then western blot proceeded with cytochrome C antibody (Biovision). The same blot was reprobed for cytochrome oxidase (subunit II), a mitochondrial membrane protein, and it confirmed that the supernatant samples were free from mitochondrial contamination.

### Tumor growth in mice

Female outbreed Balb/c mice at 4 weeks of age were purchased from Shandong Laboratory Animal Center. Seven mice were housed in wire-top cages with sawdust bedding in a clean, air-conditioned room at a temperature of 26°C and a relative humidity of 50%. 4T1 is a mammary carcinoma cell line that has been studied extensively [33]. In vivo generation of tumors was accomplished by injection of 5×10^4^ 4T1 cells suspended in 50 μl PBS into the mammary fat pad. Animal body weight and tumor size were measured and recorded. Tumor size was measured every two days in two perpendicular dimensions (a  =  length, b  =  width) with a vernier caliper, and the size recorded as a volume (mm^3^) as calculated by a * b^2^/2.

When tumors reached 8∼10 mm^2^ in size, mice were treated by intraperitoneal injection of NS, 5 mg/kg.bw and 10 mg/kg.bw fucoidan every two days. A tumor growth curve was then constructed, and data were presented as mean ± SE. After 20 days of tumor treatment, the mice were euthanized and their tumors were excised and weighed. The tumor specimens were fixed in 4% formaldehyde, embedded in paraffin, and cut in 4 μm sections for immunohistochemical analysis.

For enumeration of pulmonary metastatic nodules, the metastases appeared as discrete white nodules on the black surface of lungs insufflated and stained with a 15% solution of India ink, and then bleached by Fekette's solution.

### In situ TUNEL assay for apoptotic cells

Apoptotic cell death in paraffin-embedded tumor tissue sections was examined using the TdT-FragEL DNA fragmentation detection kit (Calbiochem, San Diego, Calf, USA) according to the manufacturer's protocol. Apoptotic cells were identified as dark brown nuclei under a light microscope. The number of apoptotic cells was counted 5 random fields (×400) in a blinded manner.

### Immunohistochemistry

Immunohistochemical analysis of VEGF expression and MVD were performed according to the procedure described elsewhere [34]. The primary antibody is monoclonal rabbit anti-murine VEGF and monoclonal rabbit anti-murine CD34 (Santa Cruz Biotechnology, Santa Cruz, CA, USA). To quantify MVD, each slide was scanned at low power magnification (×10–100). Two ‘hot spot’ areas with relatively higher number of new vessels were identified which were subsequently scanned at high power magnification (×400). Five random fields of each ‘hot pot’ area were analyzed.

### ELISA assays

The concentration of VEGF in tumor tissues was determined using mouse VEGF ELISA Kit (Jingmei Biotech, Wuhan, China) according to the manufacturer's instructions. The results of the ELISA assay in the tumor tissues were expressed as pg/mg.

### Statistical analysis

Data were expressed as the mean±S.D. Statistical analysis was performed using one-way analysis of variance (ANOVA), followed by Duncan's test, using the statistical program, SPSS11.0 for Windows. The values obtained in the assays were considered statistically different when *P*<0.05.
